# DU Is Induced by Low Levels of Urinary ATP in a Rat Model of Partial Bladder Outlet Obstruction: The Incidence of Both Events Decreases after Deobstruction

**DOI:** 10.1155/2022/6292457

**Published:** 2022-02-28

**Authors:** Luís Vale, Ana Charrua, Helena Cavaleiro, Rita Ribeiro-Oliveira, António Avelino, Tiago Antunes-Lopes, António Albino-Teixeira, Francisco Cruz

**Affiliations:** ^1^Departamento de Biomedicina, Faculdade de Medicina da Universidade Do Porto, Porto, Portugal; ^2^Serviço de Urologia, Centro Hospitalar Universitário São João, Porto, Portugal; ^3^I3S - Instituto de Investigação e Inovação Em Saúde, Universidade Do Porto, Porto, Portugal; ^4^Departamento de Cirurgia e Fisiologia, Faculdade de Medicina da Universidade Do Porto, Porto, Portugal

## Abstract

**Objectives:**

To investigate, in initial phases of bladder outlet obstruction (BOO), the urinary ATP levels, the incidence of detrusor underactivity (DU), and if they change after deobstruction.

**Methods:**

Adult female Wistar rats submitted to partial BOO (pBOO) and sham-obstruction were used. Cystometry was performed 3 or 15 days after pBOO and fluid was collected from the urethra for ATP determination. Bladders were harvested for morphological evaluation of the urothelium. DU was defined as the average of voiding contractions (VC) of sham-operated animals, with 3 SD at 15 days after the sham surgery. In another group of animals in which pBOO was relieved at 15 days and bladders were let to recover for 15 days, the incidence of DU and ATP levels were also accessed. The Kruskal–Wallis test was followed by Dunn's multiple comparisons test, and Spearman's correlation test was used.

**Results:**

DU was present in 13% and 67% of the bladders at 3 and 15 days after pBOO, respectively, and in 20% of the bladders at 15 days after deobstruction. ATP levels were significantly lower in DU/pBOO versus sham and non-DU/pBOO rats. A strong positive correlation between ATP levels and VC/min was obtained (*r* = 0.63). DU bladders had extensive areas in which umbrella cells appeared stretched, the width exceeding that presented by sham animals.

**Conclusions:**

Low urothelial ATP parallels with a high incidence of DU early after pBOO.

## 1. Introduction

Detrusor underactivity (DU) associated with prolonged partial bladder outlet obstruction (pBOO) is mostly attributed to the impairment of the detrusor muscle and the efferent pathway [1, 2]. However, effective detrusor contractions cannot be generated in the absence of sensory input arising from the bladder [3]. During bladder distension, the stretch of urothelial cells causes the release of signalling molecules among which ATP plays a key role after binding to P2X3 purinergic receptors expressed in the rich suburothelial sensory fibre network [4–6]. The disruption of this urothelial sensory fibre crosstalk decreases the frequency of expulsive bladder voiding contractions (VC) and increases the postvoid residual urine [5].

At the beginning of the obstructive process, bladders develop compensatory mechanisms to increase the strength of detrusor contraction, among which detrusor hypertrophy is the most evident [7, 8]. However, after variable periods, decompensation may supervene, resulting in weak contractions unable to initiate and maintain normal micturition [7, 8]. In the switch from compensated into decompensated stages, a decrease in blood perfusion is a constant finding in men, pigs, and rodents suffering from prolonged pBOO [4, 9]. Although ischemia may injure parasympathetic and sensory nerves, the urothelium, which has the highest metabolic rate in the whole bladder, is particularly vulnerable [2, 10].

A recent clinical study showed that 79% of men with DU and BOO submitted to prostatectomy recovered spontaneous voiding [11]. This observation seems to indicate that reversible events rather than an irreversible replacement of smooth muscle cells by collagen [7, 9] or bladder denervation [1] participate in the early development of DU. The rapid urothelial turnover makes the reactivation of the ATP-mediated urothelial sensory fibre crosstalk as one foreseeable event. With these in mind, this study tested the hypothesis that DU observed in initial phases of pBOO courses with low levels of urinary ATP and that correction of pBOO increases the proportion of animals with normal bladders and normal levels of urinary ATP.

## 2. Methods

### 2.1. Animals

All procedures were carried out under personal and project licenses approved by the Direção Geral de Veterinária (license number: 0421/000/000/2020; Lisbon, Portugal) and by the Animal Ethics Committee of Faculdade de Medicina da Universidade do Porto (FMUP) (license reference: ORBEA_ 60_2017/1218) and according to the European directive 2010/63/EU.

Female Wistar rats (3–4 months) were acquired from the FMUP animal facility and were housed in type IV cages with tap water ad libitum with an inverted light cycle, under a temperature of 22–24°C and a humidity of 45–65%.

### 2.2. Partial Bladder Outlet Obstruction and Deobstruction Procedure

pBOO was performed as described before [10], under isoflurane anaesthesia (4% for induction and 1.5% for maintenance). Briefly, after a midline suprapubic incision, the bladder neck and urethra were exposed. After the insertion of a catheter, the urethra was dissected to create a dorsal passage through which a 2–0 silk suture was passed and tied around all the urethra and a metal rod with an outside diameter of 1.0 mm. The catheter and the metal rod were removed afterwards. In control rats, the urethra was exposed and dissected as abovementioned, but no silk suture was left in place. In rat models, pBOO that lasts 6 weeks or more usually leads to a massive increment in bladder size, which does not reproduce the clinical setting. Therefore, we used 3 and 15 days as experimental time points.

For the deobstruction procedure (rBOO), the removal of the silk suture occurred on day 15, under isoflurane anaesthesia and through the previous laparotomy incision. Bladder function was investigated 15 days later.

After each surgical procedure, animals orally received 20 mg/kg of hydrochloride of tramadol, in the two subsequent days. After surgery, the animals were daily monitored. If blood was observed in the urine and animals presented 3 of the human endpoints' signs, animals would be immediately euthanized with an intraperitoneal injection of 150 mg/kg sodium pentobarbital. Two animals died before meeting these endpoints and, therefore, were not included in the experimental groups. One animal died during anaesthetic induction and the other one died 7 days after surgery of an unknown cause.

### 2.3. Cystometries and Definition of DU

The frequency of voiding contractions (VC) assessed by cystometry, in the absence of validated pressure-flow studies, has been used as a surrogate marker of DU in rats [5, 12]. For this study, the DU phenotype was defined as the average contractions of sham-operated animals minus 3 SD at 15 days after the sham surgery.

Cystometries were performed under urethane anaesthesia (1.2 g/kg body weight), with the rats lying on a heating pad with a rectal probe inserted to measure body temperature and keep it constant at 37°C (WPI ATC 1000 DC, World Precision Instruments Inc., FL, USA). The bladder was exposed through a low abdominal incision. A 20-gauge needle was inserted in the bladder dome and saline was infused (6 mL/h) using a WPI SP100IZ syringe pump (World Precision Instruments Inc., FL, USA). After a stabilization period of 30 min, the frequency of bladder VC was recorded for 10 min. Saline collected from the urethra during cystometries (spontaneous voiding or overflow) was stored at −80°C for ATP determination.

In the animals with overflow, the capacity of the detrusor muscle to generate effective VC was investigated. A 1 *µ*M acetylcholine (ACh) solution was applied in the serosa of the bladder to simulate the release of this neurotransmitter by motoneurons in the detrusor layer. Intravesical instillation of ATP (5 mg/kg) was used to simulate the effective release of ATP by the urothelium. Between these experiments, full washout was carried out.

After cystometries, while animals were still deep anaesthetised, the thoracic cavity was exposed and death was confirmed by bleeding, as described in Portuguese Decree-Law n° 1013/2013.

### 2.4. Statistical Approach and Sample Size

Our primary outcome was to determine the urinary ATP levels. Since we had more than 2 experimental groups, to analyse the data we performed the Kruskal–Wallis test followed by Dunn's multiple comparisons test, using GraphPad Prism 5.1 (GraphPad Software Inc., La Jolla, CA, USA). The results are presented as mean ± standard deviation. *P* value was used to evaluate the null hypothesis rejection/inclusion.

The number of animals used per group was calculated using the *G∗*Power 3.1.9.4 software. Taking into consideration our preliminary results, we have considered an effect size of 1.2. A *α* value of 0.05 was established, and a power of 0.95 for 4 groups (control, 3 days, 15 days, and 15 days after obstruction removal). We determined a total of 20 adult female Wistar rats (5 animals/group). However, preliminary results had shown that pBOO does not induce a systematic DU phenotype at different time points. Hence, we used 5 control animals and 30 animals (10 for each time point) submitted to BOO. These later animals were randomly divided into 3 groups: 3 days of pBOO, 15 days of pBOO, and after rBOO.

### 2.5. Histology

After cystometry, urinary bladders were harvested, fixed in formalin and embedded in paraffin. Sections of 10 *µ*m were stained with haematoxylin-eosin for histological evaluation. Urothelial width was determined using Axio Vs 40 × 64 V 4.9.1.0 software.

### 2.6. ATP Measurement

Urinary ATP levels (mol/ml) were estimated by luminometry using the ATP Bioluminescence Assay Kit HSII (Sigma, Portugal). Luciferase was added to the well, followed by an equal volume of sample. The highest value of luminescence was registered for each sample. After stabilization of the lowest luminescence reading, standard addition was performed to prevent erroneous outcomes due to any urinary compound interference.

## 3. Results

Due to experimental problems, 2 animals for analysis at 3 days after pBOO and 1 animal at 15 days after pBOO died.

### 3.1. Urodynamic Identification of the DU Phenotype after Partial BOO and after Its Removal

In sham-operated animals (*n* = 5), the mean bladder frequency, investigated at 15 days after sham surgery, was 0.6 ± 0.1 contractions per minute. The DU phenotype according to the definition was set at a bladder frequency ≤0.3 VC/min.

At 3 days after pBOO, only 13% (1/8) had the DU phenotype (0.0 ± 0.0 VC/min, voiding by overflow). At 15 days after pBOO, 67% (6/9) of the animals had a DU phenotype with 2 having 0.1 ± 0.1 VC/min and 4 voiding by overflow due to the absence of contractions.

After rBOO, only 20% (2/10) had the DU phenotype, one having a frequency of 0.1 ± 0.1 VC/min and the other voiding by overflow.

The proportion of DU animals at 15 days after pBOO was significantly higher than in the other experimental groups (chi-square analysis, *p* = 0.0198).

### 3.2. Urinary ATP Levels Associated with the Detrusor Phenotype

ATP was measurable in saline voided samples from 5 sham, 5 DU/pBOO, 6 non-DU/pBOO animals, 2 DU/rBOO, and 3 non-DU/rBOO. Due to methodological issues, the ATP in the saline voided samples of the remaining animals was not measurable.

**Figure 1:**
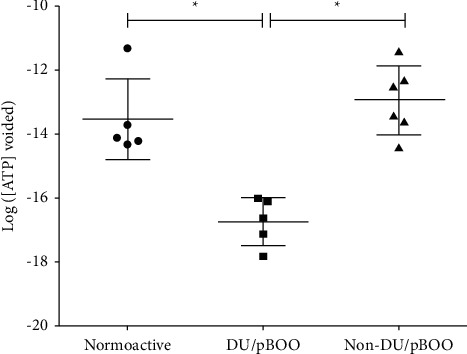
Scatter-plot of ATP levels on saline voided by animals. Scatter-plot showing the value of ATP (mol/ml) in saline voided by each animal (symbols), as well as the mean value ± standard deviation of ATP (mol/l) in saline voided by normoactive animals (sham, •), animals with DU after obstruction (DU/pBOO, ■), and animals without DU after obstruction (non-DU/pBOO, ▴).

The mean value of ATP in saline voided by sham animals (*n* = 5) was 1.4E-12 ± 2.77E-12 mol/l, while the mean value of ATP in saline voided by non-DU/pBOO animals (*n* = 6) was 7.20E-13 ± 1.43E-12 mol/l. The mean value of ATP in saline voided by DU/pBOO animals (*n* = 5) was 4.18E-17 ± 3.79E-17 mol/l. The comparison of these three groups using the Kruskal–Wallis test showed that they are statistically different (*p* < 0.002). Dunn's multiple comparisons test showed that the mean level of ATP in saline voided by the DU/pBOO group was lower than the mean level of ATP in saline voided by sham (*p* < 0.05; Figure 1) and lower than the mean level of ATP in saline voided by non-DU/pBOO (*p* < 0.05; Figure 1). Also, the mean level of ATP in saline voided by sham and non-DU/pBOO were similar (*p* > 0.05; Figure 1).

After deobstructed, the mean value of ATP in saline voided by animals with DU/rBOO (*n* = 2) was 2.03E-19 ± 1.75E-19 mol/l. In contrast, the mean value of ATP in saline voided by animals without DU/rBOO (*n* = 3) was several thousand times higher, 1.72E-12 ± 1.94E-12 mol/l, similar to the mean value of ATP in saline voided by sham rats (*p*=0.25).

**Figure 2:**
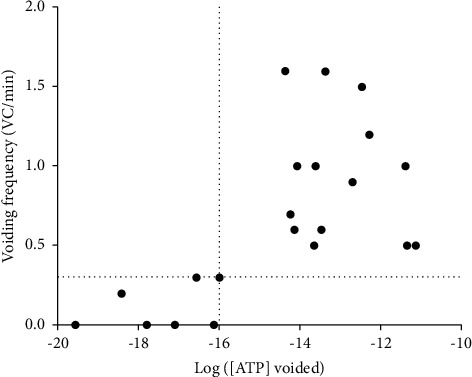
Spearman's correlation between log ([ATP] voided) levels and voiding frequency (VC/min). Scatter plot showing Spearman's correlation between the logarithm of the amount of saline-voided ATP and the voided contraction presented by each animal (•). All values on or below the horizontal dash line represent animals with the DU phenotype. The DU/pBOO and DU/rBOO animals all presented levels of saline-voided ATP inferior to 1.00E-16 (values on the left side of the dash line vertical line). VC, voiding contractions.

A Spearman's correlation test showed a strong positive correlation between ATP levels and VC/min (*r* = 0.63; *p*=0.0022) (Figure 2). A cutoff of 1.00E-16 mol/ml of ATP distinguished DU from non-DU animals.

### 3.3. Assessment of Detrusor Contractility in Animals with DU and Overflow

**Figure 3:**
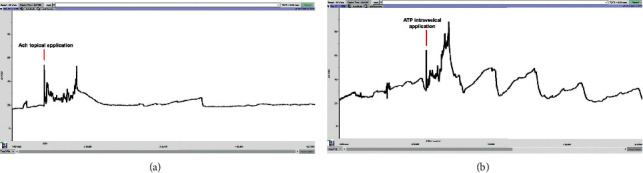
Typical cystometrograms of DU animals after stimulation with ACh and ATP. Images of a typical cystometrogram of one of the six animals that did not present bladder contraction (voided by overflow) and received (a) a topical application of 1 *µ*M of ACh in the serosa. In this cystometrogram, the single application of ACh (red arrow) generated an immediate voiding contraction. (b) After washout, the same animal intravesical received 5 mg/kg of ATP (red arrow), resulting in voiding contractions.

In bladders with the overflow DU phenotype (1 animal at day 3 after pBOO, 4 animals at day 15 after pBOO, and 1 animal after rBOO), the contractile capacity of the detrusor was investigated. Topical serosal application of ACh (1 *µ*M) solution generated immediate expulsive detrusor contractions allowing an almost complete emptying of the bladder. After washout, intravesical application of ATP (5 mg/kg) generated immediate expulsive detrusor contractions (representative cystometrograms in Figure 3). Detrusor contractions induced by the application of ACh and ATP occurred in all animals with DU in overflow indicating that detrusor smooth muscle cells maintained contractile capability.

### 3.4. Histological Evaluation of the Urothelium in Animals with DU

**Figure 4:**
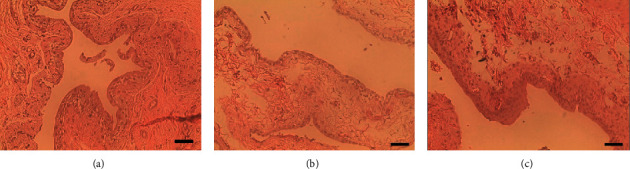
Representative microphotographies of bladder urothelium. Representative microphotographies of bladder slides stained with H&E of (a) an animal with a normal phenotype and (b, c) of an animal with the DU phenotype. (a, c) Normal urothelial cell features. The microphotography (b), however, presents an extra-stretched urothelial cell configuration. Scale bar: 50 *µ*m.

The urinary bladders of DU animals (both after pBOO and rBOO) presented areas with stretched, flattened umbrella cells with a width of 37.1 ± 6.6 *µ*m among areas of urothelium where the normal width was maintained (12.3 ± 2.0 *µ*m; Figure 4). The width of the umbrella cells in stretched areas was statistically different from the ones presented by non-DU animals (Kruskal–Wallis test, *p*=0.0015 and Dunn's multiple comparisons test, a mean rank difference of 18.25).

## 4. Discussion

The DU/pBOO phenotype was associated with low levels of urinary ATP, therefore confirming our first hypothesis that in the early phases of pBOO, DU is associated with low urinary ATP levels. The DU/rBOO phenotype was also associated with low urinary levels of ATP, while the non-DU/rBOO phenotype had ATP levels similar to those of sham animals. Moreover, a strong correlation was detected between ATP levels and bladder VC. Therefore, DU induced by pBOO is due, at least in part, to an inadequate generation of ATP-initiated sensory stimuli. As ATP detected in the urine is most likely released from umbrella cells, their abnormal morphology in DU bladders agrees with the reduction of urinary ATP [4].

Studies carried out in men validate this interpretation. Cho et al. investigated the changes in the levels of ATP in urothelial biopsies of men with DU and benign prostatic hyperplasia and concluded that the neurotransmitter was significantly decreased [13]. Also, Jiang and Kuo observed that patients with chronic BOO had a lower expression of P2X3 receptors [14]. The mechanism leading to a decrease of urinary levels of ATP in our animals may be explained by cyclic events of ischemia and reperfusion and the consequent accumulation of free oxygen radicals [9, 15]. A high expression of the hypoxic-inducible factor was demonstrated in partially obstructed bladder mice [16] and in human bladder with prostatic-induced BOO [17]. As mentioned before, the bladder layer more susceptible to ischemia is the urothelium that has a metabolic rate threefold higher than that of the detrusor [18].

This study showed that at 3 days of partial chronic BOO, DU could already be detected, although most of the animals still had bladder voiding contractions. These observations are in accordance with the concept that, to overcome obstruction, bladders develop swift compensatory mechanisms. Examples include the increase of nerve growth factor levels in the bladder wall [19] that enhances the excitability of bladder sensory C-fibers, including those that activate the sacral micturition reflex. Smooth muscle cells may develop abnormal shapes like ultraclose abutments and protrusion cell junctions that facilitate cell-to-cell propagation of contractile waves [20]. In addition, supersensitivity to acetylcholine may appear due to impairment of cholinergic innervation [21].

In the animals let to survive 15 days with pBOO, the predominant urodynamic phenotype, observed in 67% of the animals, evolved to a low frequency of bladder contractions or even to an absence of contractions [2, 15]. The hypothesis that DU was caused by a severe impairment of detrusor smooth muscle was, however, excluded in the study. In fact, the animals at overflow were subjected to detrusor smooth muscle stimulation through the application of ACh to the serosa to simulate more closely the effect of the release of these neurotransmitters from parasympathetic nerve endings. In addition, ATP was instilled into the bladder cavity to simulate the urothelial ATP crosstalk with the P2X3 receptors in suburothelial sensory fibres, which activates the micturition reflex [4, 5]. In both cases, prompted detrusor contractions were generated, indicating the contractile ability of the smooth muscle cells.

After rBOO, only 20% of the animals had a DU phenotype. This proportion contrasts with that of the 67% observed in animals 15 days after pBOO. These observations suggest a remarkable capacity of the rat bladder to recover the voiding function, a characteristic that may be attributed, in part, to the turnover of the urothelial layer [4] and the return of ATP availability. Non-DU/rBOO animals had urinary ATP levels similar to sham rats. These experimental observations fit well with a recent clinical report that showed that prostatectomy led to the reappearance of bladder voiding contractions in the majority of men with DU due to prostatic obstruction [11].

Not all patients with BOO will develop DU [22]. Therefore, a critical aspect in the management of BOO is to avoid a state of irreversible bladder damage. In fact, patients with severe ultrastructural changes in the morphology of the smooth muscle cells rarely report a successful outcome after prostatectomy [23]. Hence, early detection of the appearance of irreversible changes may play a key role. Gheneini and coworkers have shown characteristic changes in mRNAs and miRNAs expressed in male detrusor samples [24]. However, these biomarkers require invasive biopsies to collect detrusor fragments. A simpler biomarker able to detect if urothelial cell disability leads to bladder functional impairment due to BOO may be the ATP measured in the urine. In fact, in men with BOO and detrusor overactivity, there is a positive correlation between urinary ATP and voided volume [25]. By analogy, it might be expected that low ATP levels may indicate the DU phenotype. At this moment, the complexity of ATP measurement is an obvious drawback.

The study has some limitations. Ideally, we should have been able to follow the same animals throughout the experimental procedure and perform awake cystometries to characterize their urodynamic phenotype and urinary ATP throughout the entire experiment. That would have required the placement of a catheter in the bladder at the very beginning. Unfortunately, in preliminary experiments, too many animals developed bladder dehiscence after the creation of pBOO, which led us to abandon such an experimental design.

## Data Availability

The data used to support the findings of this study are available from the corresponding author upon request.
